# Baltic Sea ecosystem-based management under climate change: Integrating social and ecological perspectives

**DOI:** 10.1007/s13280-015-0669-1

**Published:** 2015-05-28

**Authors:** Erik Bonsdorff, Agneta Andersson, Ragnar Elmgren

**Affiliations:** Faculty of Science and Engineering, Environmental and Marine Biology, Åbo Akademi University, FI-20500 Turku, Finland; Department of Ecology and Environmental Science, Umeå University, 901 87 Umeå, Sweden; Department of Ecology, Environment and Plant Sciences, Stockholm University, 106 91 Stockholm, Sweden

**Fig. 1:**
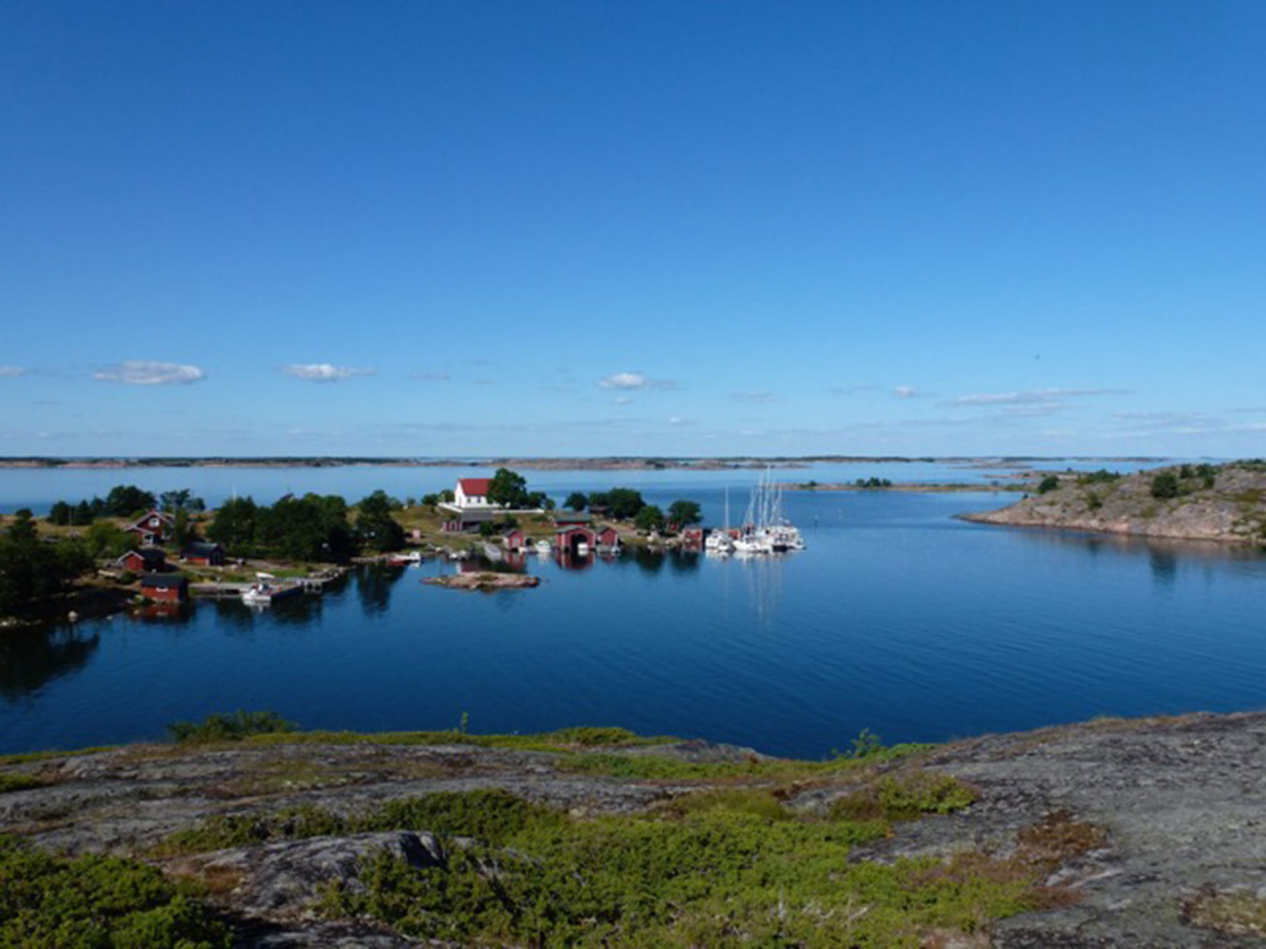
The extensive archipelago areas of the Baltic Sea provide valuable ecosystem services to the coastal inhabitants, where local small-scale fishery meets tourism and recreation (Photo: Erik Bonsdorff)

The Baltic Sea is a shallow, semi-enclosed sea in northern Europe, and the second largest brackish-water area in the world, after the Black Sea. It is surrounded by industrialized nations, and has a catchment that is four times its sea area, with a large riverine outflow to the sea. Its southern and eastern half harbors have extensive areas of intensive agriculture. The Baltic Sea is also a highly valued resource for the human population of roughly 85 million living around it (Fig. 1), but the ecosystem services it provides have been compromised in recent decades by deteriorated water quality, primarily due to eutrophication, exploitation of coastal areas, overfishing, and contamination by toxic pollutants. These problems are all caused by human activities, and international political structures have been created to manage them. The Helsinki Convention, governed by the Helsinki Commission (HELCOM), handles environmental pollution, while fisheries since 2005 are governed through agreements between the EU, in accordance with its Common Fisheries Policy, and Russia, with advice from the International Council for the Exploration of the Sea (ICES). Since its various problems interact extensively, there is general agreement that the Baltic Sea needs a common and ecosystem-based management of its marine environment.

Interdisciplinary research is essential to provide relevant science in support of ecosystem-based management. In 2009, the Swedish government decided to stimulate science in Sweden by offering increased research funding for universities, to be distributed to clearly specified research areas based on applications with a 5-year research plan. In the field of Strategic Marine Environmental Research, two applications were funded: “The Baltic Ecosystem Adaptive Management” (BEAM) program from Stockholm University; and the program “Ecosystem dynamics in the Baltic Sea in a changing climate perspective” (ECOCHANGE) from Umeå University, in cooperation with Kalmar University College (now Linnaeus University). The BEAM program has funded interdisciplinary research in support of ecosystem-based management of the Baltic Sea area—the ECOCHANGE program research on the effects of climate change on the Baltic Sea. The two programs have cooperated in areas of joint interest, and this *AMBIO* Special Issue reports a selection of results from the two programs. See http://www.su.se/ostersjocentrum/english/beam/publications for other publications from BEAM and http://www.umf.umu.se/english/ecochange/publications for more ECOCHANGE publications. Both programs have invested in research aimed at improving our knowledge of the social–ecological system of the Baltic Sea, and especially the interactions among the major problem areas, such as nutrient enrichment, contaminant loads, overfishing, invasive species, and climate change.

For the purpose of planning and editing this special issue, the two programs created a guest editorial board, and invited Professor Erik Bonsdorff of ÅboAkademi University to serve as Guest Editor. In addition to the authors, the editorial board has included, from Stockholm University, Thorsten Blenckner, Elena Gorokhova, and Nastassja Åstrand Capetillo; from Umeå University, Terry Bidleman, Kristina Viklund and Johan Wikner; and from Linnaeus University, Catherine Legrand. We thank them and all the contributing authors for their diligent work to keep this issue within time and space limits, while striving for high scientific quality, and *AMBIO’s* editor-in-chief Bo Söderström for helpful advice during this process.

